# Cumulative behavioral and metabolic determinants of health are associated with higher inflammation-related indices: insights from a cross-sectional study (NHANES 2005–2018)

**DOI:** 10.3389/fpubh.2025.1602629

**Published:** 2025-06-19

**Authors:** Anzhi Wang, Youping Zeng, Xiaoyan Gao, Xunge Lin, Shenshen Du, Ping Wang, Yun Pan

**Affiliations:** ^1^The First Affiliated Hospital of Jinan University, Guangzhou, China; ^2^Department of Emergency, Zhujiang Hospital, Southern Medical University, Guangzhou, China

**Keywords:** behavioral determinants of health, metabolic determinants of health, systemic immune-inflammation index, systemic inflammatory response index, NHANES

## Abstract

**Objective:**

This study innovatively investigates the cumulative associations between behavioral determinants of health (BDoH), metabolic determinants of health (MDoH), and systemic inflammation biomarkers in U.S. adults, using a novel cross-sectional framework to quantify their synergistic effects.

**Methods:**

Utilizing cross-sectional data from 18,500 participants in the National Health and Nutrition Examination Survey (NHANES 2005–2018 cycle), we developed a composite exposure model integrating BDoH (smoking status, physical activity, dietary quality) and MDoH (obesity metrics, hypertension, diabetes) through standardized questionnaires and clinical measurements. Systemic immune-inflammation index (SII) and systemic inflammatory response index (SIRI) were calculated from peripheral blood cell counts. Multivariable-adjusted logistic regression models examined dose–response relationships, with trend analysis explicitly testing cumulative BDoH-MDoH interactions.

**Results:**

The cohort (mean age 44.3 ± 0.3 years; 52.4% male) demonstrated significant positive associations between adverse health determinants and inflammatory indices. Current tobacco use (OR = 1.32, 95%CI = 1.18–1.47), suboptimal diet (HEI < 52: OR = 1.24, 95%CI = 1.11–1.38), obesity (BMI ≥ 30 kg/m^2^: OR = 1.41, 95%CI = 1.27–1.56), and central adipometry (OR = 1.39, 95%CI = 1.25–1.54) showed strongest correlations with elevated SII/SIRI. Metabolic disorders exhibited distinct patterns: hypertension and diabetes associated specifically with SIRI elevation (OR = 1.19, 95%CI = 1.06–1.33 and OR = 1.17, 95%CI = 1.03–1.32, respectively), while physical inactivity (<600 MET-min/week) uniquely correlated with SII increase (OR = 1.26, 95%CI = 1.13–1.40). Notably, our cumulative model revealed synergistic effects: exposure to ≥3 adverse behavioral determinants amplified inflammation risks (SII: OR = 1.57, 95%CI = 1.42–1.73; SIRI: OR = 1.49, 95%CI = 1.35–1.64), with significant dose-dependent trends (P-trend<0.001). Co-occurring metabolic abnormalities demonstrated additive inflammatory effects (P-trend<0.001), exceeding individual risk factor impacts.

**Conclusion:**

This cross-sectional study provides the first evidence that integrated BDoH-MDoH cumulative exposure models uncover distinct and synergistic inflammatory pathways. Both individual and combined behavioral-metabolic risk factors significantly associate with systemic inflammation biomarker elevation, highlighting the necessity of dual-target intervention strategies.

## 1 Introduction

The influence of behavioral and metabolic factors on health has attracted significant attention. Current research indicates that behavioral risk factors are significantly associated with an increased risk of death from cardiovascular disease (CVD) (1). There is growing literature that behavioral and metabolic determinants of health (BDoH, MDoH) affect systematic inflammation level, however the exact nature of these relationships remains largest undefined. The systemic immune-inflammation index (SII) is a novel marker of inflammation derived from the counts of neutrophils, platelets, and lymphocytes. Initially employed for evaluating the prognosis of hepatocellular carcinoma (2). Subsequently, the SII has also been verified to be linked to metabolic syndrome, diabetic nephropathy, cerebrovascular disease, depression in diabetes, and osteoporosis in postmenopausal women (3–8). The Systemic Inflammatory Response Index (SIRI) is another inflammatory marker based on the counts of neutrophils (Neut), monocytes (Mono), and lymphocytes (Lymph) in peripheral blood, which is used to assess the systemic inflammatory status of the body. Likewise, SIRI was originally employed for forecasting the prognosis of pancreatic cancer and was linked to unfavorable outcomes (9). Recently, SIRI has been recognized as an indicator of the chronic inflammatory status of the human body, demonstrating a J-shaped correlation with all-cause mortality and cardiovascular disease mortality in hypertensive patients (10). While SII and SIRI shared numerous similarities in assessing cancer prognosis and evaluating the prognosis of patients with cardiovascular diseases (2, 9, 11), distinctions persist between the two. Studies indicated that in the population aged <60 years, a higher SIRI was linked to an increased incidence of myocardial infarction, while SII did not show any association (11). Dziedzic EA et al. (12) affirmed the association of SIRI with the diagnosis of ST-segment elevation myocardial infarction in acute coronary syndrome patients. The predictive value of SIRI for stroke incidence in the asthma population (AUC = 0.618) surpassed that of SII (AUC = 0.552) (13), and it also demonstrated superior predictive capabilities for both all-cause mortality and cardiovascular disease mortality in the obese population (14). Consequently, the combined use of SII and SIRI provides a more comprehensive and objective assessment of systemic inflammation than either index alone, serving as valuable auxiliary tools for quantifying inflammatory status.

Behavioral factors are closely related to inflammation. Consumption of a high-fat diet, smoking, and chronic insomnia has been linked to elevated levels of pro-inflammatory markers C-reactive protein (CRP) and IL-6 (15–18). Conversely, higher dietary fiber intake has been linked to reduced SII, and physical activity has been shown to markedly decrease SII in pediatric cancer patients (19, 20). In addition, metabolic factors are also intricately linked to inflammation. For example, obesity was characterized by systemic low-grade chronic inflammation, marked by elevated levels of CRP, IL-6, IL-8, and tumor necrosis factor-*α* (TNF-α) in the serum (21, 22). Moreover, a higher SIRI is closely related to obesity in the older adult (23). After accounting for metabolic risk factors, the cardiovascular disease mortality hazard ratio declined from 1.54 to 1.34 (1).

While existing studies on behavioral-metabolic risk factors and inflammatory indices offer clinical insights, their limitations stem from complex inter-plays among risk factors. This study systematically examines SII/SIRI associations by quantifying cumulative effects through composite exposure models while deciphering behavioral-metabolic interactions, thereby elucidating synergistic inflammatory effects that surpass individual factor impacts. Our findings aim to provide actionable guidance for clinical prevention and advance personalized medicine approaches by bridging current knowledge gaps in risk factor inter-dependencies.

## 2 Methods

### 2.1 Study participants

The study’s analysis data was derived from National Health and Nutrition Examination Survey (NHANES), which is a comprehensive survey based on the entire United States (the U.S.) population, aimed at collecting and assessing health and nutritional status data for both adults and children in 15 unincorporated counties in the U.S. Since 1999, NHANES has been updating its data every 2 years, utilizing complex, stratified, multistage probability sampling techniques applied by the Centers for Disease Control and Prevention (CDC) to survey nationally representative samples of residents, which are then made freely accessible to the public.

**Figure 1:**
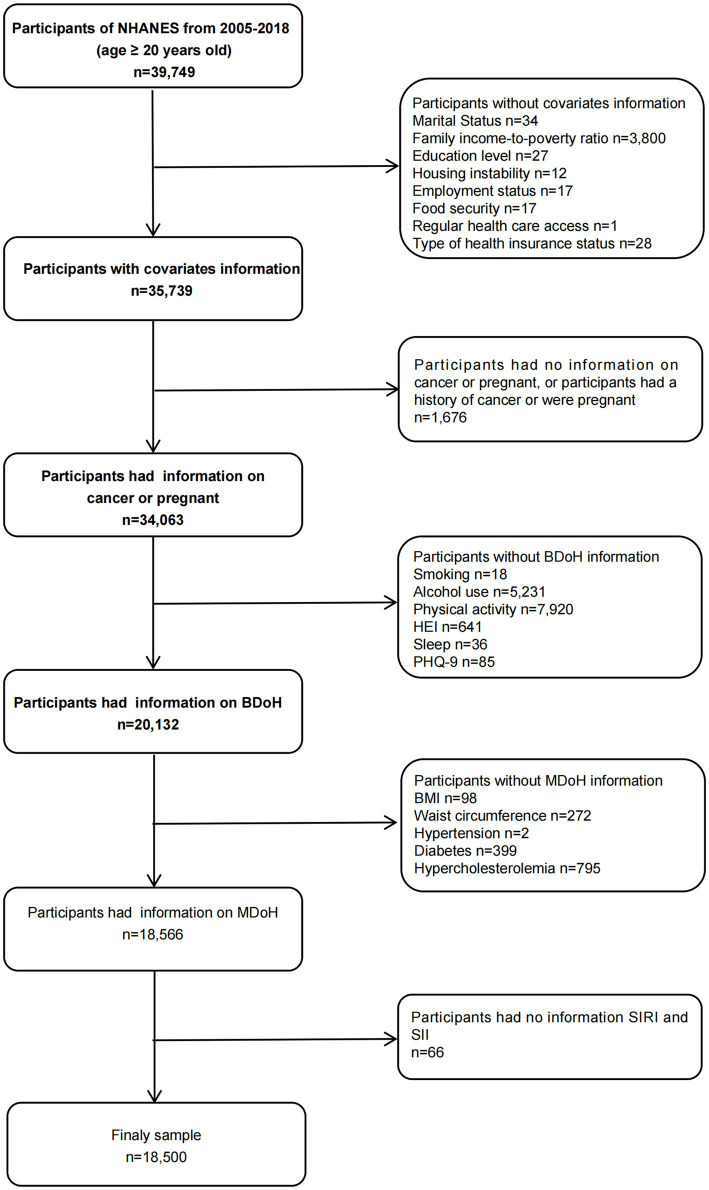
Flow chart of the screening of eligible participants.

This study utilized a sample of adults aged 20 and over in the U.S. from 2005 to 2018, spanning across 10 cycles. During the sample selection, the following criteria were excluded: (1) individuals lacking covariate data; (2) participants with no recorded BDoH and MDoH information; (3) pregnant or cancer patients; (4) participants with no SIRI and SII data (Figure 1). Finally, a total of 18,500 individuals were included in the analysis. The NHANES protocol was approved by the National Center for Health Statistics Institutional Review Board and written informed consent was obtained from each participant prior to data collection. The detailed methods and data were obtained from the official website https://www.cdc.gov/nchs/nhanes/index.htm.

### 2.2 Data collection

During each two-year survey cycle, all participants are required to complete household interviews and surveys at the examination center, undergo physical examinations and blood sampling, and were sent to the central laboratory for the analysis of relevant indicators.

### 2.3 Inflammation-related index SIRI and SII

In this study, the peripheral blood samples of the participants were collected by trained staff and analyzed using the Mobile Examination Center (MECs) automatic analyzer (Beckman Coulter MAXM) for lymphocyte, neutrophil, monocyte, and platelet counts (all ×10^3 cells/μmL) (2). Subsequently, we calculated the SII and SIRI using the following formulas and categorized the participants into low inflammatory index groups (SIRI < 1.02, SII < 467.96) and high inflammatory index groups (SIRI ≥ 1.02, SII ≥ 467.96) based on the weighted median calculations (SIRI = 1.02, SII = 467.96) (2, 9).


$$\text{SIRI}=\frac{\left(\text{Neutrophil count}\times \text{monocyte count}\right)}{\text{lymphocyte count}}$$

$$\mathrm{SII}=\frac{\left(\text{Platelet count}\times \text{neutrophil count}\right)}{\text{lymphocyte count}}$$

### 2.4 Behavioral determinants of health(BDoH)

Upon reviewing the NHANES questionnaire, we systematically identified and classified all BDoH variables associated with the participants.

#### 2.4.1 Smoking

During household interviews, smoking is categorized based on self-reports from adults aged 20 and above as follows: former smoker: >100 cigarettes in a lifetime, but currently not smoking; never smoker: <100 cigarettes in a lifetime; current smoker: ≥100 cigarettes in a lifetime, smoking occasionally or daily. We assigned a value of 1 for current smoking and 0 for former or never smoking (24).

#### 2.4.2 Alcohol use

Alcohol intake is categorized into five groups: never drinking alcohol (less than 12 cups in a lifetime), light drinking (men ≤ 2 cups per day, women ≤ 1 cup per day), moderate drinking (men ≤ 3 and > 2 cups per day, women ≤ 2 and > 1 cup per day, or binge drinking for ≥2 days but <5 days per month), heavy drinking (men ≥ 4 cups per day, women ≥ 3 cups per day, or binge drinking for ≥5 days per month), former drinking (did not drink in the past year, but consumed ≥12 cups within a year or ≥ 12 cups throughout life) (25). We designated light, moderate, and heavy drinking as 1, and former or never drinking as 0 (26).

#### 2.4.3 Physical activity

The Global Physical Activity Questionnaire was used to collect data, which was then analyzed based on the World Health Organization’s guidelines. The metabolic equivalent (MET) of physical activities (PA) is calculated using a formula that considers the types, frequency, and duration of activities within different work areas, transportation, and leisure time. The calculated physical activity is expressed in MET-minutes per week, indicating moderate to vigorous physical activity. In accordance with the guidelines for adult physical activity, meeting (≥600 MET-minutes/week) is classified as 0, while not meeting the criteria (<600 MET/min/week) is classified as 1.

#### 2.4.4 Diet

HEI is a comprehensive assessment of individual dietary patterns based on the 2015 “Dietary Guidelines for Americans.” It involves participants recalling all food and beverages consumed in a day every 3 to 10 days and calculating the total nutrient intake and 13 dietary components, consisting of 9 adequacy components and 4 moderation components. The adequacy components include total fruit, whole fruit, total vegetables, vegetables and legumes, whole grains, dairy, total protein foods, seafood and plant proteins, as well as fatty acids (the ratio of unsaturated to saturated fatty acids) (27, 28). The moderation components include refined grains, sodium, added sugars, and saturated fats. Subsequently, each component undergoes Scott-Rao chi-squared test and weighted logistic regression to derive the final score for HEI-2015, ranging from 0 to 100. A higher score denotes better overall dietary quality. The median score for participants’ HEI was 52, with a score less than 52 classified as 1, and a score of 52 or more as 0.

#### 2.4.5 Sleep

We categorized the duration of sleep during workday nights: a sleep duration of 6–8 h per day will be denoted as 0, while a sleep duration of less than 6 h or more than 8 h per day will be denoted as 1.

#### 2.4.6 Emotion

Participants’ depressive state is measured using the Patient Health Questionnaire (PHQ-9) (29). This self-report tool is well-validated (Cronbach’s *α* = 0.89) for assessing depressive symptoms over the past 2 weeks. It consists of 9 items, each scored from 0 to 3, resulting in a total score ranging from 0 to 27. The PHQ-9 is used to evaluate the severity of depressive symptoms in participants of the survey: a total score >4 is categorized as emotional abnormality and assigned a value of 1, whereas a total score ≤4 is assigned a value of 0 for emotional normality.

### 2.5 Metabolic determinants of health(MDoH)

Specific MDoH include body mass index (BMI), central obesity, hypertension, diabetes, and hypercholesterolemia. We assigned a score of 1 for each metabolic risk factor present, and 0 for the absence of metabolic risk factors.

BMI is calculated as weight (in kg) divided by the square of height (in meters) (kg/m^2^). Individuals are classified as (BMI < 30 kg/m^2^) and obese(BMI ≥ 30 kg/m^2^) groups (30).

Central obesity is defined as a waist circumference of at least 102 cm for men and at least 88 cm for women.

Hypertension is diagnosed by a doctor or the use of antihypertensive medication, with an average systolic blood pressure >140 mmHg and/or diastolic blood pressure >90 mmHg.

Diabetes is diagnosed by a doctor and the use of medication or insulin, defined by a fasting blood sugar level of at least 7 mmol/L (≥126 mg/dL) and a hemoglobin A1c level of at least 6.5%.

A total cholesterol to high-density lipoprotein cholesterol ratio (Total–HDL cholesterol ratio) ≥ 5 is considered indicative of the risk of hypercholesterolemia.

### 2.6 Covariates

The specific classification criteria for covariates were as follows: Age (years); Sex: female, male; Race: non-Hispanic White, non-Hispanic Black, Mexican American, Other; Cardiovascular disease (CVD): whether the participants have been diagnosed by a medical professional with congestive heart failure, coronary heart disease, angina pectoris, myocardial infarction, or stroke. If any these conditions were confirmed, they were classified as having CVD; Education Level: high school graduate or more, less than high school education; Marital Status: married or living with a partner, not married nor living with a partner; Family income-to-poverty ratio: the Poverty Income Ratio (PIR) was calculated by dividing household income by the poverty line for a specific family size; The PIR was categorized into two groups: PIR < 300% was indicative of poverty; while a PIR ≥ 300% indicated no poverty issue; Housing instability: the participants owned the residence they live in or not; Employment status: by asking participants about their job activities over the past week, those who were not working are classified as unemployed, while the rest could be classified as employed, students and retires; Food security: The situation of food insecurity was evaluated using the Adult Food Security Survey Module of the United States Department of Agriculture. The U.S. Department of Agriculture’s Adult Food Security Survey Module is a validated survey and is regarded as the gold standard for measuring food insecurity in the United States. Since the data collection cycle from 2005 to 2006, all households have been asked these questions and no income filters have been used. Based on the responses of families to these surveys, the NHANES literature Outlines the classification of food security status according to the number of affirmative responses in the adult module: 0 affirmative responses indicate full food security, 1–2 affirmative responses indicate marginal food security, 3–5 affirmative responses indicate a low food security, and 6–10 affirmative responses indicate a very low food security. Consistent with the established research, this study classified individuals with full food security group, and combined the marginal, low, or very low food security into other group (31). Regular health care access: participants were divided into those with one or more regular health care facilities and the rest were without routine place for health care or emergency room visits; type of health insurance status: individuals with private insurance, the other is government insurance or none.

### 2.7 Statistical analysis

**Figure 2:**
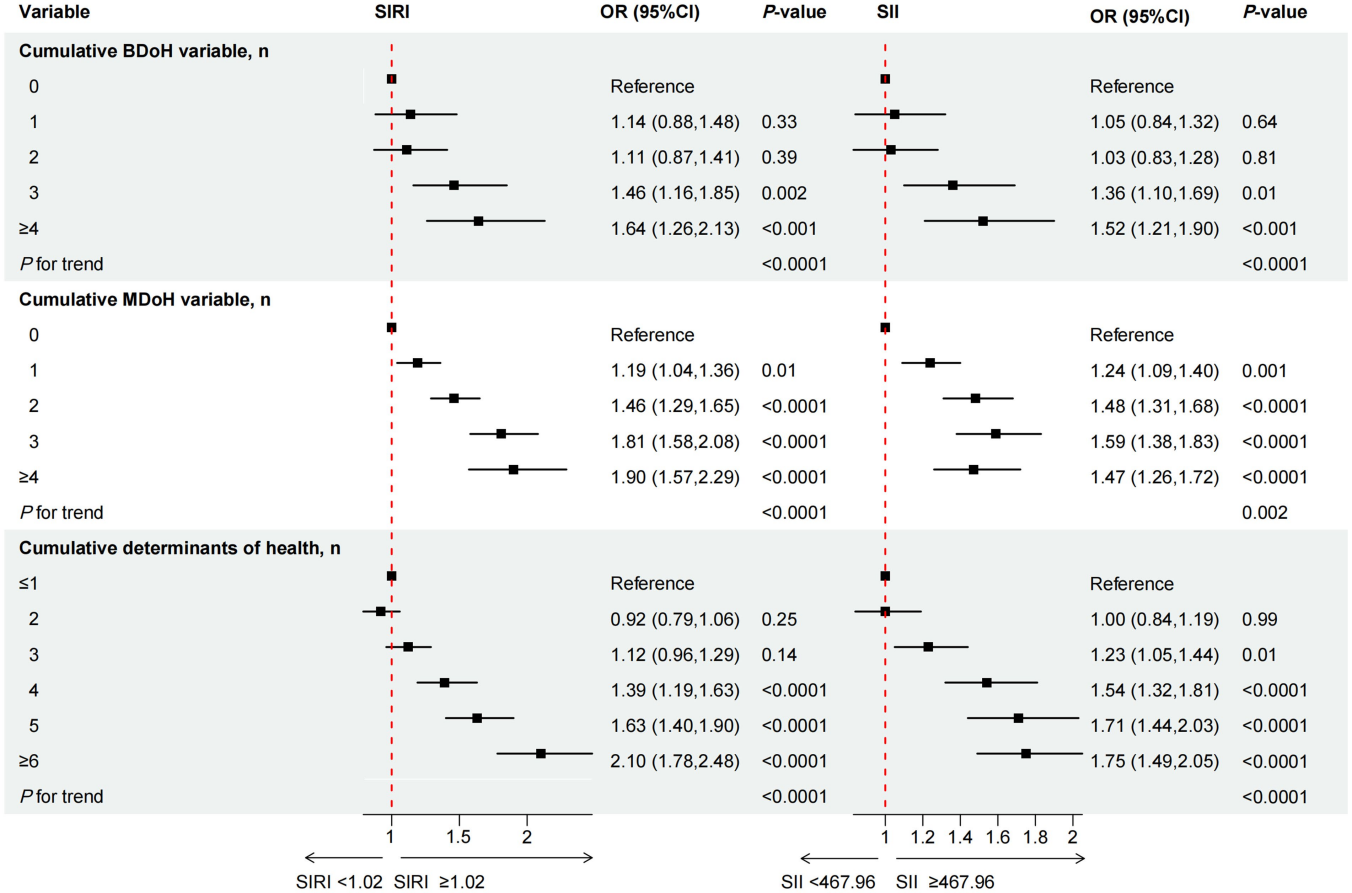
The association between cumulative behavioral and metabolic determinants of health and the inflammation-related index SIRI and SII. OR of SIRI and SII, by the number of behavioral determinants of health, adjustment for age, sex, race/ethnicity, CVD, education level, marital status, family income-to-poverty ratio, housing instability, employment, food security, regular health care access, type of health insurance, and all other metabolic determinants of health listed in the Table 2; OR of SIRI and SII, by the number of behavioral determinants of health, adjustment for age, sex, race/ethnicity, CVD, education level, marital status, family income-to-poverty ratio, housing instability, employment, food security, regular health care access, type of health insurance, and all other behavioral determinants of health.

**Figure 3:**
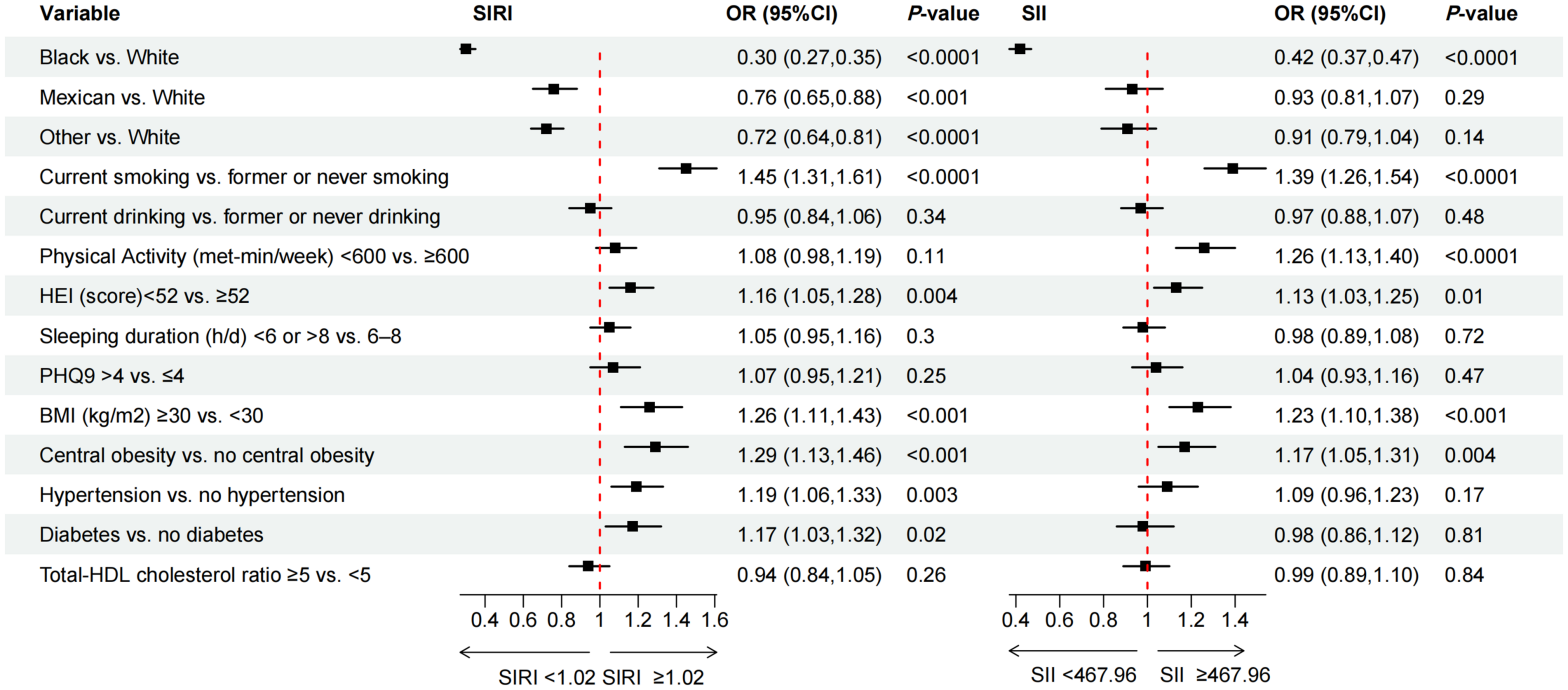
The association between specific behavioral and metabolic determinants of health and the inflammation-related index SIRI and SII. OR of SIRI and SII, by the specific behavioral and metabolic determinants of health, adjustment for age, sex, race/ethnicity, CVD, education level, marital status, family income-to-poverty ratio, housing instability, employment, food security, regular health care access, type of health insurance.

We utilized the NHANES database’s recommended weights to enhance the representativeness of the data for the entire American population. Chi-square (χ2) and one-way analysis of variance (ANOVA) tests were performed in Tables 1, 2 to compare demographic characteristics of SIRI and SII across various levels. Continuous variable results are presented as mean ± standard error (SE), and binary or categorical variables are displayed using frequency counts and proportions. After adjusting the covariates related to the exposure and outcome variables, we constructed a series of weighted multivariable logistic regression models to explore the odds ratios (ORs) and 95% confidence intervals (CIs) between SIRI and SII and cumulative health behaviors and metabolic determinants. Subsequently, we further employed multivariable logistic regression to examine the specific relationships between determinants and inflammatory index. To demonstrate the robustness of the results, we also utilized subgroup analyses and interaction tests to assess the relationships between health behavioral and metabolic determinants and inflammatory index in different populations. The covariates related to exposure and outcome variables were adjusted in the aforementioned models. Please refer to the notes in Tables 1–4, Figures 2, 3 for note details. To account for multiple comparisons across behavioral (BDoH) and metabolic (MDoH) determinants and inflammatory outcomes (SII/SIRI), we applied the Benjamini-Hochberg false discovery rate (FDR) correction with a threshold of *q* < 0.05 for all statistical tests. Finally, the proportions of binary inflammatory markers in cumulative health behaviors and metabolic determinants were visualized using line graphs. All analyses were conducted using R software version 4.3.2, with statistical significance defined as a two-tailed *p*-value less than 0.05.

**Table 1:** Characteristics of participants with full samples.

Variable	Overall	SIRI		*p*-value	SII		*p*-value
		<1.02	≥1.021.02		<467.96	≥467.96	
Age, years old	44.31 ± 0.28	43.03 ± 0.31	45.59 ± 0.32	<0.0001	43.75 ± 0.34	44.87 ± 0.31	< 0.001
Sex, *n* (%)				<0.0001			<0.0001
Female	8,609 (47.63)	4,937 (54.51)	3,672 (45.49)		4,139 (45.78)	4,470 (54.22)	
Male	9,891 (52.37)	4,763 (46.08)	5,128 (53.92)		5,538 (53.87)	4,353 (46.13)	
Race, *n* (%)				<0.0001			<0.0001
Non-Hispanic White	8,222 (69.33)	3,545 (46.12)	4,677 (53.88)		3,798 (47.53)	4,424 (52.47)	
Non-Hispanic Black	3,770 (10.09)	2,593 (69.65)	1,177 (30.35)		2,491 (65.62)	1,279 (34.38)	
Mexican American	2,836 (8.22)	1,469 (52.16)	1,367 (47.84)		1,406 (49.98)	1,430 (50.02)	
Other	3,672 (12.36)	2093 (55.10)	1,579 (44.90)		1982 (51.26)	1,690 (48.74)	
CVD, *n* (%)				<0.0001			0.02
No	17,147 (94.28)	9,194 (51.14)	7,953 (48.86)		9,037 (50.31)	8,110 (49.69)	
Yes	1,353 (5.72)	506 (33.00)	847 (67.00)		640 (45.27)	713 (54.73)	
Education level, *n* (%)				0.66			0.4
High school graduate or more	14,832 (87.62)	7,814 (50.17)	7,018 (49.83)		7,769 (50.16)	7,063 (49.84)	
Less than high school education	3,668 (12.38)	1886 (49.56)	1782 (50.44)		1908 (49.01)	1760 (50.99)	
Marital status, *n* (%)				0.001			0.01
Married or living with a partner	11,256 (63.11)	5,960 (51.28)	5,296 (48.72)		5,967 (51.10)	5,289 (48.90)	
Not married nor living with a partner	7,244 (36.89)	3,740 (48.07)	3,504 (51.93)		3,710 (48.16)	3,534 (51.84)	
Family income-to-poverty ratio, *n* (%)				0.72			0.95
≥ 300%	7,511 (53.10)	4,074 (50.27)	3,437 (49.73)		3,974 (50.05)	3,537 (49.95)	
< 300%	10,989 (46.90)	5,626 (49.91)	5,363 (50.09)		5,703 (49.98)	5,286 (50.02)	
Housing instability, *n* (%)				0.002			0.002
Own home	11,365 (67.60)	5,857 (48.94)	5,508 (51.06)		5,845 (48.81)	5,520 (51.19)	
Rent or other arrangement	7,135 (32.40)	3,843 (52.52)	3,292 (47.48)		3,832 (52.53)	3,303 (47.47)	
Employment, *n* (%)				0.03			0.002
Employed, student, retired	14,921 (82.94)	7,758 (49.57)	7,163 (50.43)		7,896 (50.71)	7,025 (49.29)	
Not employed	3,579 (17.06)	1942 (52.67)	1,637 (47.33)		1781 (46.63)	1798 (53.37)	
Food security, *n* (%)				0.52			0.37
Full food security	12,804 (77.12)	6,746 (50.25)	6,058 (49.75)		6,713 (50.29)	6,091 (49.71)	
Marginal, low, or very low	5,696 (22.88)	2,954 (49.59)	2,742 (50.41)		2,964 (49.11)	2,732 (50.89)	
Regular health care access, *n* (%)				0.67			0.04
≥One regular health care facility	13,903 (77.64)	7,331 (50.22)	6,572 (49.78)		7,206 (49.39)	6,697 (50.61)	
None or emergency room	4,597 (22.36)	2,369 (49.67)	2,228 (50.33)		2,471 (52.18)	2,126 (47.82)	
Type of health insurance, *n* (%)				0.02			0.64
Private insurance	10,288 (65.40)	5,512 (50.88)	4,776 (49.12)		5,402 (50.17)	4,886 (49.83)	
Government or none	8,212 (34.60)	4,188 (48.62)	4,024 (51.38)		4,275 (49.73)	3,937 (50.27)	

Continuous variables were showed as mean ±SE, categorical variables were showed as frequency (percentage). CVD: cardiovascular disease.

**Table 2:** Behavioral, metabolic determinants of health among study participants, by SIRI and SII median.

Variable	Overall	SIRI		*p*-value	SII		*p-*value
		<1.02	≥1.021.02		<467.96	≥467.96	
Behavioral determinants of health							
Smoke status, *n* (%)				<0.0001mes New Roman			<0.0001
Never or former	14,539 (79.34)	7,923 (51.97)	6,616 (48.03)		7,780 (51.49)	6,759 (48.51)	
Now	3,961 (20.66)	1777 (42.93)	2,184 (57.07)		1897 (44.36)	2064 (55.64)	
Alcohol use, *n* (%)				0.53			0.23
Never or former	4,732 (20.39)	2,516 (49.42)	2,216 (50.58)		2,483 (48.90)	2,249 (51.10)	
Now	13,768 (79.61)	7,184 (50.27)	6,584 (49.73)		7,194 (50.30)	6,574 (49.70)	
Physical activity (met-min/week), *n* (%)				0.12			< 0.0001
≥600	14,199 (77.75)	7,459 (50.52)	6,740 (49.48)		7,610 (51.57)	6,589 (48.43)	
<600	4,301 (22.25)	2,241 (48.64)	2060 (51.36)		2067 (44.59)	2,234 (55.41)	
HEI (score), *n* (%)				< 0.0001			0.002
≥52	8,312 (46.03)	4,550 (52.85)	3,762 (47.15)		4,502 (51.98)	3,810 (48.02)	
<52	10,188 (53.97)	5,150 (47.76)	5,038 (52.24)		5,175 (48.34)	5,013 (51.66)	
Sleep time (h), *n* (%)				0.14			0.58
6–8	13,825 (76.62)	7,275 (50.51)	6,550 (49.49)		7,234 (50.17)	6,591 (49.83)	
<6 or >8	4,675 (23.38)	2,425 (48.76)	2,250 (51.24)		2,443 (49.51)	2,232 (50.49)	
PHQ9 (score), *n* (%)				0.01			0.002
≤4	14,421 (79.29)	7,673 (50.87)	6,748 (49.13)		7,686 (50.90)	6,735 (49.10)	
>4	4,079 (20.71)	2027 (47.14)	2052 (52.86)		1991 (46.65)	2088 (53.35)	
Cumulative BDoH variable, *n* (%)				< 0.0001			< 0.0001
0	935 (4.15)	549 (55.98)	386 (44.02)		532 (53.43)	403 (46.57)	
1	4,413 (24.92)	2,427 (52.27)	1986 (47.73)		2,427 (52.33)	1,986 (47.67)	
2	6,167 (34.21)	3,340 (52.90)	2,827 (47.10)		3,332 (53.19)	2,835 (46.81)	
3	4,425 (23.31)	2,174 (46.26)	2,251 (53.74)		2,186 (46.15)	2,239 (53.85)	
≥4	2,560 (13.41)	1,210 (43.77)	1,350 (56.23)		1,200 (43.29)	1,360 (56.71)	
Metabolic determinants of health determinants of health							
BMI (kg/m2), *n* (%)				< 0.0001			< 0.0001
<30	11,783 (65.06)	6,464 (53.39)	5,319 (46.61)		6,422 (52.57)	5,361 (47.43)	
≥30	6,717 (34.94)	3,236 (43.96)	3,481 (56.04)		3,255 (45.26)	3,462 (54.74)	
Central obesity, *n* (%)				< 0.0001			< 0.0001
No	8,754 (47.66)	4,918 (55.05)	3,836 (44.95)		4,950 (54.64)	3,804 (45.36)	
Yes	9,746 (52.34)	4,782 (45.59)	4,964 (54.41)		4,727 (45.81)	5,019 (54.19)	
Hypertension, *n* (%)				< 0.0001			0.002
No	11,752 (67.43)	6,499 (53.14)	5,253 (46.86)		6,284 (51.30)	5,468 (48.70)	
Yes	6,748 (32.57)	3,201 (43.80)	3,547 (56.20)		3,393 (47.35)	3,355 (52.65)	
Diabetes				< 0.0001			0.1
No	15,824 (89.74)	8,440 (51.03)	7,384 (48.97)		8,291 (50.28)	7,533 (49.72)	
Yes	2,676 (10.26)	1,260 (41.92)	1,416 (58.08)		1,386 (47.74)	1,290 (52.26)	
Total–HDL cholesterol ratio, *n* (%)				< 0.0001			0.26
<5	14,963 (81.13)	7,940 (51.03)	7,023 (48.97)		7,873 (50.27)	7,090 (49.73)	
≥5	3,537 (18.87)	1760 (46.09)	1777 (53.91)		1804 (48.95)	1733 (51.05)	
Cumulative MDoH variable, *n* (%)				< 0.0001			< 0.0001
0	5,369 (31.58)	3,140 (57.42)	2,229 (42.58)		3,081 (56.10)	2,288 (43.90)	
1	3,980 (21.84)	2,138 (51.61)	1842 (48.39)		2,123 (50.32)	1857 (49.68)	
2	4,145 (22.16)	2,113 (48.11)	2032 (51.89)		2034 (45.95)	2,111 (54.05)	
3	3,152 (16.18)	1,495 (41.71)	1,657 (58.29)		1,532 (44.79)	1,620 (55.21)	
≥4	1854 (8.24)	814 (39.86)	1,040 (60.14)		907 (47.12)	947 (52.88)	
Cumulative determinants of health, *n* (%)				< 0.0001			< 0.0001
≤1	1847 (11.17)	1,129 (56.94)	718 (43.06)		1,094 (56.75)	753 (43.25)	
2	3,032 (17.46)	1788 (58.79)	1,244 (41.21)		1774 (57.29)	1,258 (42.71)	
3	3,700 (19.74)	2048 (54.14)	1,652 (45.86)		2015 (52.53)	1,685 (47.47)	
4	3,551 (19.34)	1787 (48.31)	1764 (51.69)		1764 (46.72)	1787 (53.28)	
5	2,950 (15.71)	1,433 (44.56)	1,517 (55.44)		1,416 (44.43)	1,534 (55.57)	
≥6	3,420 (16.59)	1,515 (38.87)	1905 (61.13)		1,614 (43.98)	1806 (56.02)	

Categorical variables were showed as frequency (percentage). HEI, healthy eating index; PHQ-9, Patient Health Questionnaire-9.

**Table 3:** Subgroup analysis and interaction of the association between cumulative behavioral and metabolic determinants of health and SIRI.

SIRI	Cumulative BDoH variable					*P* for interaction
	0	1	2	3	4	
	Reference	OR (95%CI)	OR (95%CI)	OR (95%CI)	OR (95%CI)	
Subgroups						
Age, years old						0.87
<65	Reference	1.18 (0.85, 1.64)	1.12 (0.83, 1.52)	1.44 (1.08, 1.93)	1.60 (1.18, 2.19)	
≥65	Reference	0.95 (0.63, 1.44)	1.00 (0.67, 1.48)	1.44 (0.94, 2.20)	1.48 (0.89, 2.46)	
Sex						0.77
Female	Reference	1.13 (0.81, 1.56)	1.06 (0.78, 1.44)	1.28 (0.90, 1.83)	1.53 (1.09, 2.14)	
Male	Reference	1.15 (0.77, 1.73)	1.14 (0.77, 1.67)	1.57 (1.08, 2.28)	1.61 (1.04, 2.47)	
Race						0.26
White	Reference	1.18 (0.83, 1.68)	1.15 (0.83, 1.60)	1.59 (1.16, 2.18)	1.72 (1.21, 2.44)	
Non-White	Reference	1.13 (0.86, 1.49)	1.03 (0.80, 1.33)	1.20 (0.90, 1.59)	1.40 (1.03, 1.90)	
CVD						0.49
No	Reference	1.13 (0.86, 1.47)	1.08 (0.85, 1.38)	1.40 (1.10, 1.78)	1.58 (1.22, 2.05)	
Yes	Reference	1.61 (0.67, 3.88)	1.52 (0.68, 3.39)	2.08 (0.83, 5.24)	1.63 (0.64, 4.16)	

SIRI	Cumulative MDoH variable					*P* for interaction
	0	1	2	3	4	
	Reference	OR (95%CI)	OR (95%CI)	OR (95%CI)	OR (95%CI)	
Subgroups						
Age, years old						0.01
<65	Reference	1.17 (1.03, 1.33)	1.49 (1.32, 1.69)	1.86 (1.62, 2.13)	1.79 (1.50, 2.13)	
≥65	Reference	1.81 (1.21, 2.68)	1.62 (1.10, 2.40)	2.02 (1.36, 2.99)	3.11 (1.96, 4.93)	
Sex						0.01
Female	Reference	1.12 (0.92, 1.37)	1.34 (1.12, 1.61)	1.72 (1.39, 2.12)	1.46 (1.12, 1.88)	
Male	Reference	1.28 (1.09, 1.49)	1.59 (1.34, 1.88)	1.92 (1.63, 2.26)	2.37 (1.87, 2.99)	
Race						0.16
White	Reference	1.23 (1.04, 1.47)	1.45 (1.24, 1.70)	1.90 (1.58, 2.29)	2.08 (1.60, 2.70)	
Non-White	Reference	1.12 (0.97, 1.29)	1.47 (1.28, 1.68)	1.62 (1.36, 1.92)	1.57 (1.29, 1.90)	
CVD						0.8
No	Reference	1.20 (1.06, 1.37)	1.46 (1.30, 1.65)	1.83 (1.59, 2.09)	1.86 (1.55, 2.24)	
Yes	Reference	1.39 (0.73, 2.63)	1.87 (1.07, 3.29)	2.20 (1.20, 4.02)	2.93 (1.58, 5.44)	

Adjusted for covariates other than themselves, such as age, sex, race/ethnicity, CVD, education level, marital status, family income-to-poverty ratio, housing instability, employment, food security, regular health care access, type of health insurance.

**Table 4:** Subgroup analysis and interaction of the association between cumulative behavioral and metabolic determinants of health and SII.

SII	Cumulative BDoH variable					*P* for interaction
	0	1	2	3	4	
	Reference	OR (95%CI)	OR (95%CI)	OR (95%CI)	OR (95%CI)	
Subgroups						
Age, years old						0.15
<65	Reference	1.05 (0.80, 1.36)	0.95 (0.73, 1.24)	1.25 (0.97, 1.61)	1.37 (1.04, 1.79)	
≥65	Reference	0.89 (0.59, 1.35)	1.20 (0.80, 1.80)	1.52 (0.94, 2.43)	1.80 (0.91, 3.55)	
Sex						0.58
Female	Reference	0.88 (0.67, 1.14)	0.87 (0.66, 1.14)	1.14 (0.86, 1.50)	1.24 (0.93, 1.65)	
Male	Reference	1.23 (0.86, 1.77)	1.15 (0.81, 1.64)	1.49 (1.07, 2.08)	1.63 (1.13, 2.36)	
Race						0.15
White	Reference	1.04 (0.76, 1.43)	0.99 (0.73, 1.34)	1.41 (1.04, 1.91)	1.47 (1.08, 2.00)	
Non-White	Reference	1.06 (0.83, 1.36)	1.05 (0.82, 1.34)	1.15 (0.90, 1.47)	1.39 (1.07, 1.80)	
CVD						0.81
No	Reference	1.07 (0.85, 1.33)	1.03 (0.82, 1.28)	1.34 (1.08, 1.67)	1.46 (1.16, 1.84)	
Yes	Reference	0.78 (0.35, 1.75)	0.75 (0.36, 1.56)	0.97 (0.45, 2.09)	1.17 (0.51, 2.70)	

SII	Cumulative MDoH variable					*P* for interaction
	0	1	2	3	4	
	Reference	OR (95%CI)	OR (95%CI)	OR (95%CI)	OR (95%CI)	
Subgroups						
Age, years old						0.05
<65	Reference	1.20 (1.05, 1.38)	1.50 (1.32, 1.72)	1.64 (1.42, 1.90)	1.44 (1.23, 1.69)	
≥65	Reference	1.40 (0.94, 2.09)	1.24 (0.83, 1.85)	1.20 (0.84, 1.72)	1.35 (0.88, 2.06)	
Sex						0.84
Female	Reference	1.23 (1.03, 1.47)	1.50 (1.28, 1.76)	1.49 (1.22, 1.82)	1.47 (1.14, 1.89)	
Male	Reference	1.23 (1.04, 1.46)	1.41 (1.20, 1.65)	1.61 (1.34, 1.94)	1.41 (1.15, 1.72)	
Race						0.26
White	Reference	1.25 (1.06, 1.47)	1.42 (1.20, 1.68)	1.64 (1.35, 2.00)	1.43 (1.17, 1.73)	
Non-White	Reference	1.18 (1.01, 1.37)	1.53 (1.32, 1.78)	1.38 (1.20, 1.58)	1.44 (1.17, 1.77)	
CVD						0.19
No	Reference	1.24 (1.09, 1.41)	1.48 (1.30, 1.68)	1.61 (1.40, 1.85)	1.48 (1.26, 1.74)	
Yes	Reference	0.93 (0.53, 1.64)	1.07 (0.56, 2.05)	0.84 (0.47, 1.49)	0.83 (0.45, 1.53)	

Adjusted for covariates other than themselves, such as age, sex, race/ethnicity, CVD, education level, marital status, family income-to-poverty ratio, housing instability, employment, food security, regular health care access, type of health insurance.

## 3 Results

### 3.1 Participant characteristics

In this cross-sectional study based on the NHANES (2005–2018), we included a total of 18,500 adults aged ≥20 years, representing a population of 91,127,127 in the U.S. In Table 1, we categorized participants into groups with lower and higher inflammatory-related indices based on the median values of the SIRI and SII (median SIRI = 1.02, median SII = 467.96), and presented the clinical characteristics of the participants. Their average age was 44.31 ± 0.28 years. The group with higher levels of SIRI was more likely to be male, non-Hispanic white, have cardiovascular disease, not married nor living with a partner, own a home, have a job or be a student or retired and with government insurance or none. On the other hand, the group with higher levels of SII was more likely to be female, non-Hispanic white, have cardiovascular disease, not married nor living with a partner, own a home, be unemployed and have at least one regular health care facility. Significant differences in SIRI and SII levels were observed across different ages, genders, races, presence of cardiovascular disease, marital status, fixed residence, and employment status.

### 3.2 Characteristics of healthy behavioral and metabolic risk factors in participants

Table 2 displays participant characteristics by SIRI/SII quartiles, including dose–response distributions of the six behavioral and five metabolic determinants (both individually and in combination). In the group with higher SIRI levels, there was a higher proportion of current smokers, poor diet (HEI < 52), and PHQ9 > 4, as well as a higher accumulation of behavioral risk factors. In addition, regardless of the type of metabolic risk factor, they accounted for more than half of the group with a higher SIRI. Similarly, the group with higher SII, in addition to the specific BDoH mentioned above, also had 55.41% of participants with PA < 600 (met-min/week). In terms of MDoH, the group with higher SII had a higher prevalence of participants with hypertension and a Total–HDL cholesterol ratio.

### 3.3 The relationship between cumulative BDoH and MDoH and the inflammation-related index SIRI and SII

The relationship between cumulative BDoH and MDoH and the inflammation-related index SIRI and SII was illustrated in Figure 2. We adjusted for covariates that may affect the outcome variables, for specific adjustments please refer to the notes in Figure 2. Individuals exhibiting an accumulation of ≥3 BDoH were found to be associated with higher levels of SIRI and SII compared to those without, and the probability increased as the accumulation quantity grew (*P* for trend < 0.05). Similarly, participants with one or more risk factors showed higher levels of SIRI or SII compared to those without cumulative MDoH, *P* for trend < 0.05. When the two types of determinants were added together, it was also found that the cumulative risk factors were significantly positively associated with the inflammation-related indices.

### 3.4 The relationship between race, specific BDoH and MDoH and the inflammation-related index

In addition to investigating the correlation between cumulative health determinants and inflammation-related indices, we also conducted analysis of the association between specific determinants and exposure variables. Figure 3 illustrated the association between race, specific BDoH and MDoH with inflammation-related indices. To mitigate the impact of confounding factors on the results, we concurrently adjusted the covariates. For further details, please refer to the note section. The results of the multivariable logistic regression analysis indicated that, non-white individuals were associated with lower SIRI compared to white individuals. Additionally, current smoker, poor diet (HEI < 52), BMI ≥ 30, central obesity, hypertension, and diabetes were significantly positively associated with higher SIRI. Only black individuals exhibited a significant negative correlation with SII. Among other risk factors, current smokers, PA < 600 (met-min/week), poor diet (HEI < 52), BMI ≥ 30, and individuals with central obesity showed a significant positive association with higher SII, demonstrating statistical differences.

### 3.5 Subgroup analysis and interaction testing

In order to assess the robustness of the association between BDoH and MDoH with the inflammation-related index SIRI and SII across varied age, gender, race, and CVD populations, subgroup analyses were conducted (refer to Tables 3, 4). The findings indicated that among individuals aged ≥65 years and with CVD showed no significant statistical disparity between SIRI and behavioral risk factors, whereas consistent positive correlations were observed in other populations. This implies a link between the accumulation of behavioral and metabolic risk factors and an increase in SIRI, with age and MDoH jointly influencing the participants’ SIRI (refer to Table 3). Table 4 similarly depicted the relationship between BDoH and MDoH and SII in unique populations. Specifically, the outcomes showed that, except for participants aged ≥65 years, female, and those with CVD, SII exhibited a substantial positive correlation with behavioral risk factors; additionally, SII displayed a notable positive correlation with metabolic risk factors, except for participants aged ≥65 years and those with CVD. Notably, no significant interactions were observed across the different subgroups in Table 4.

### 3.6 The status of inflammation-related index within varying accumulative quantities of behavioral and metabolic determinants among the participants

**Figure 4:**
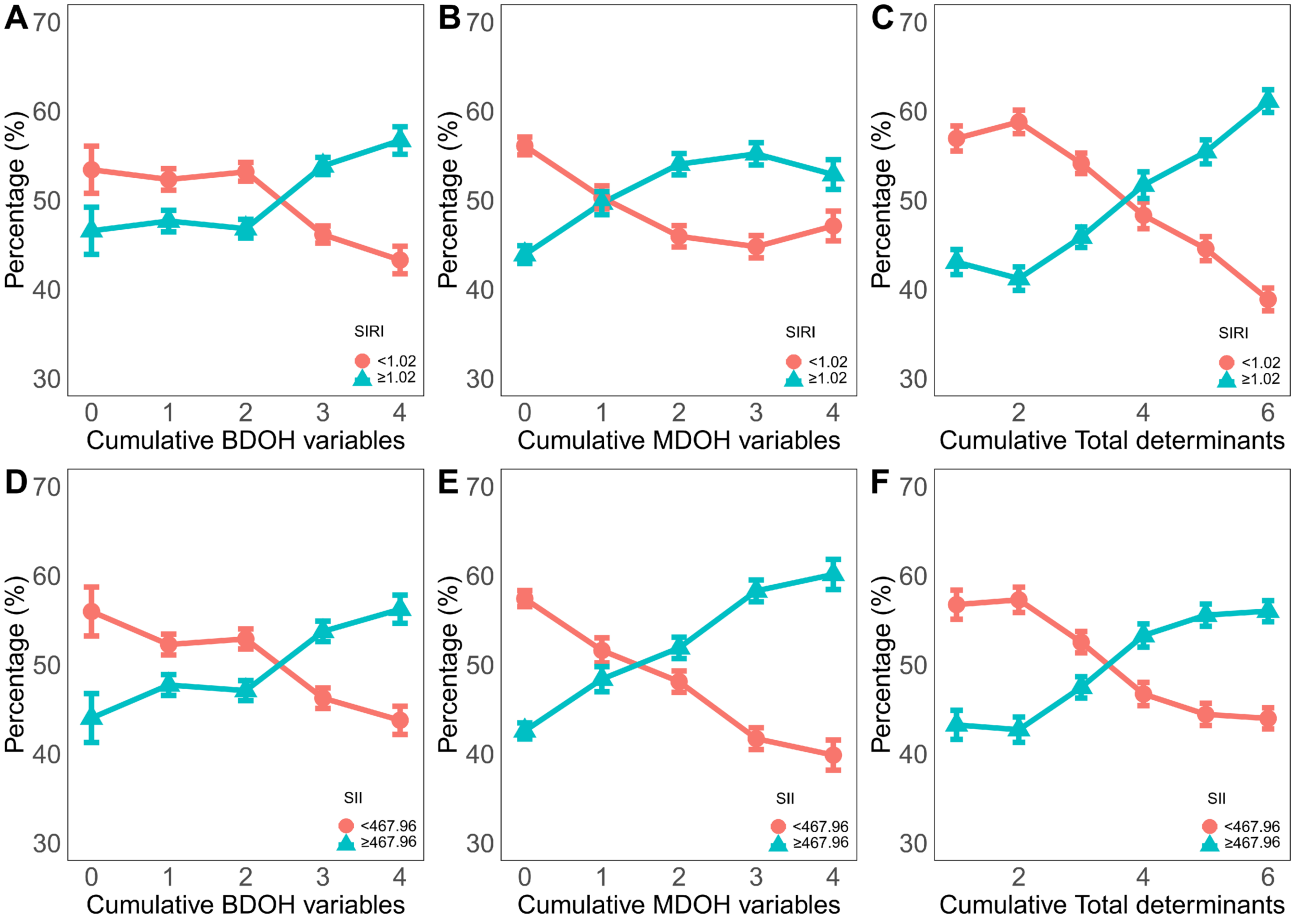
The proportion of the dichotomous variable of the inflammatory index among the different cumulative behavioral and metabolic determinants of health. **(A-C)** the circles represent SIRI<1.02 cutoff value, and the triangles represent SIRI> or =1.02 cutoff value. **(D-F)** the circles represent the cutoff value of SII<467.96, and the triangles represent the cutoff value of SII> or =467.96.

A higher cumulative number of BDoH and MDoH was associated with an increased number of individuals with elevated levels of SIRI and SII in Figure 4. In addition, the proportion of individuals with an elevated inflammation index peaked in scenarios with the highest accumulation of risk factors.

## 4 Discussion

The study indicated that unfavourable BDoH (including current smoking, HEI < 52) and MDoH (such as BMI ≥ 30, central obesity) were associated with higher levels of SIRI and SII.

SII and SIRI are novel inflammation markers derived from three subsets of white blood cells and platelets, effectively reflecting the body’s inflammatory response and immune status. Previous research has established a close association between specific behavioral and metabolic risk factors and SII and SIRI (19, 23). In our analysis of a large sample of 18,500 participants, we observed significant positive correlations between smoking, poor diet (HEI < 52), BMI ≥ 30, central obesity, and elevated levels of SIRI and SII. It is crucial to maintain the balance between pro-inflammatory and anti-inflammatory processes for immune homeostasis (32, 33). Smoking has been found to simultaneously regulate both pro-inflammatory and anti-inflammatory processes (34). Previous research suggested that smoking not only elevates the production of pro-inflammatory factors such as TNF-*α*, IL-1, IL-6, IL-8, and granulocyte-macrophage colony stimulating factor (GM-CSF), but also diminished the levels of the anti-inflammatory cytokine IL-10 (35). Studies by McEvoy and colleagues have revealed that both current and former smokers demonstrate higher levels of hs-CRP and IL-6 compared to non-smokers (36). This may be attributed to the substantial release of reactive oxygen species from cigarettes, which activated inflammatory genes like IL-8 and TNF-α within epithelial cells, resulting in the secretion of inflammatory mediators that ultimately fostered the recruitment of chronic immune cells and an inflammatory response (37). The above content preliminarily revealed the potential mechanism of higher levels of SIRI and SII in current smokers.

Regarding food safety, studies have shown that insecurity food and malnutrition has been linked to heightened risk factors for cardiovascular diseases (38). Chronic inflammation is a common pathological feature in numerous cardiovascular diseases (39). Our findings indicated that poor diet (HEI < 52) is positively associated with elevated SIRI and SII. This preliminary finding indicated that insecurity food might be associated with cardiovascular diseases through the pro-inflammatory pathways of the immune system, although further research is needed to elucidate the specific mechanisms. In contrast, plant-based diets, including the Mediterranean and Dietary Approaches to Stop Hypertension (DASH), exhibited a negative correlation with oxidative stress and pro-inflammatory biomarkers, potentially leading to a decrease in biomarkers associated with inflammation and immune response, such as CRP, IL-6, and TNF-*α* (40). The reason for this lay in the presence of compounds like carotenoids and flavonoids in plant-based diets may directly or indirectly regulate inflammation and immune processes (41).

A study conducted on the Swiss population found that obese individuals exhibited elevated levels of hypersensitive C-reactive protein (hs-CRP), whereas those with abdominal obesity showed increased levels of TNF-α. Subsequent stratified analysis showed a positive correlation between TNF-α levels and waist circumference in men, and between TNF-α levels and BMI in women (42). Research related to the increase in CRP and IL-6 levels associated with obesity also validated the existence of immune cell infiltration in the obese population (43). Consistent with previous studies, our findings suggested a positive correlation between BMI ≥ 30, central obesity, and elevated levels of SIRI and SII. This was probably due to obese patients experiencing prolonged low-level systemic inflammation, leading to the infiltration of immune cells into local white adipose tissue, including macrophages and lymphocytes (44).

Furthermore, our findings indicated a significant positive correlation between high levels of SIRI and hypertension and diabetes, while no association with SII. Earlier research has demonstrated the superiority of SIRI over SII in diagnosing and predicting mortality in cardiovascular diseases (11, 14). Because hypertension and diabetes were significant risk factors for cardiovascular diseases, these patients might have had concurrent cardiovascular conditions, leading to distinctions between the two. Regarding physical activity, our findings indicated that PA < 600 (met-min/week) was solely positively associated with high levels of SII, with no correlation with SIRI. This aligned with previous studies demonstrating that exercise could markedly decrease SII levels in pediatric cancer patients (20). For individuals with elevated SII, physical activity could decrease the overall mortality rate by 30% and the risk of cardiovascular disease-related deaths by 32% in the Chinese participant cohort (45). This validated the anti-inflammatory impact of persistent exercise, yet its mechanism remained unclear.

Although the investigation of the relationship between specific behavioral and metabolic risk factors, and inflammation-related indices has provided some clinical reference value, it is important to acknowledge its notable limitations due to the intricate and numerous inherent interactions among human risk factors. A meta-analysis demonstrated that high levels of moderate-intensity physical activity could counteract the elevated risk of mortality associated with sedentary behavior, confirming the interplay among different behavioral factors (46). Our research innovatively explored the overall correlation between cumulative BDoH and MDoH and the inflammation-related index SIRI and SII, especially concerning the cumulative effects of risk factors. Our results demonstrated that multiple risk factors exert a cumulative impact on the inflammatory indices, with the cumulative effect values escalating alongside the increase in risk factors. Likewise, among American adults, the presence of six or more adverse Social Determinants of Health (SDoH) was associated with approximately eightfold increased risk of premature death (47). Another study also identified a positive correlation between the quantity of social, behavioral, and metabolic risk factors and the risk ratio for cardiovascular disease mortality (1). Hence, we asserted that investigating the influence of cumulative risk factors on the inflammation-related indices might more accurately depict the actual physiological state and hold greater clinical significance than analyzing single risk factors. In comparison to prior studies, our research objectively elucidated the correlation between cumulative BDoH and MDoH and the inflammation-related indices.

Our study has several strengths. Firstly, it utilized samples from a nationally representative large-scale cohort, representing a population of 91,127,127 in the United States. Secondly, we focused on the overall behaviors and metabolic risk factors, examining their correlations with the inflammatory index SII and SIRI, particularly the cumulative effects of these risk factors. Our study encompassed populations with multiple risk factors, thus providing deeper insights. Lastly, our sensitivity analysis revealed that the association between behavioral and metabolic risk factors and SIRI and SII remained stable, thus indicating the robustness of the study results.

Of course, this study also has some limitations. First, the behavioral and metabolic risk factors in NHANES were collected in a cross-sectional manner, so we could not determine the causal relationship between these risk factors and SIRI and SII. Second, the participants in NHANES might not have represented the global population, so we needed further verification of its applicability to other ethnicities. In addition, there are too many potential covariates that may affect SIRI and SII, such as infection factors, differences in medical conditions, etc. This study cannot completely rule out the potential influencing variables. While we analyzed cumulative effects, future studies could explore non-linearity or critical thresholds using spline models or piecewise regression. Finally, it should be noted that using binary variables for BDoH significantly reduces the sensitivity and granularity of the results. We strongly recommend that future studies employ continuous or ordinal measurements of BDoH to better capture nuanced effects.

## 5 Conclusion

In summary, unfavourable BDoH and MDoH were associated with higher SIRI and SII. Moreover, adverse determinants of health exert a cumulative influence on the inflammation-related indices. While this observational study does not establish causality, improving lifestyle and controlling modifiable metabolic factors may have the potential to reduce inflammation levels and lower the risk of inflammation-related diseases. These findings are anticipated to offer more precise and effective guidance for health management and disease prevention, as well as to pave the way for new approaches to personalized medical care and health interventions.

## Data Availability

The original contributions presented in the study are included in the article/supplementary material, further inquiries can be directed to the corresponding authors.
